# The concept, transformation logic, practical dilemmas, and countermeasures in promoting the transformation of smart-empowered older adult care services via New Quality Productive Forces

**DOI:** 10.3389/fpubh.2025.1560539

**Published:** 2025-05-20

**Authors:** Liping Fu, Meng Li, Lanping He, Ya’nan Fang

**Affiliations:** ^1^College of Management and Economics, Tianjin University, Tianjin, China; ^2^Tianjin Renai College, Tianjin, China; ^3^Tianjin Administration Institute, Tianjin, China

**Keywords:** digitalization, New Quality Productive Forces, smart-empowered older adult care service systems, transformation logic, digital transformation

## Abstract

Digitization is gradually becoming integrated into the living contexts of older adults, especially in China. The New Quality Productive Forces (NQPF), one of the tools for the promotion of digitalization, is the key driver in promoting the digital transformation of older adult care services. Through the extensive review, this study analyzes the effects of NQPF on the development of smart-empowered older adult care service systems. The results reveal that the digital transformation of older adult care services is driven by the interactive effects of scientific and technological innovations, digital cognition, and the service systems. In addition, the transformation logic reveals how the older adult care industry upgrades through a multi-subject coordinated model of the supply-and-demand dimensions under New Quality Productive Forces. This study offers valuable references and insights into the transformation of digital older adult care services.

## 1 Introduction

Recently, owing to diverse living aspirations and higher-quality requirements of older adult care, traditional older adult care service systems have been unable to fulfill the care demands of an increasing number of older adult people in China, Europe, the United States, and other countries globally. As new-generation innovative information technologies, such as artificial intelligence (AI) and cloud computing, are increasingly being applied to diverse fields in contemporary society, the digital and intelligent transformation of the industry is gradually becoming an important method for improving the quality of older adult care services (OACS) in China. During the inspection period in Heilongjiang Province in 2023, the Chairman of China introduced a new theory of New Quality Productive Forces (NQPF), which stresses the continuous integration of scientific and technological innovation resources and increases the development of strategic emerging and future industries to accelerate its formation (1). Scientific and technological innovation is the core factor of NQPF and the foundation for developing smart-empowered older adult care service system (SOACSS). Through diverse digital technologies, SOACSS can not only eliminate the limitation of space and time to the greatest extent and improve the accessibility of OACS but also address the shortage of the older adult care workforce caused by the aging population. Hence, a comprehensive and in-depth understanding of the logical relationship between the NQPF and SOACSS is crucial.

Furthermore, understanding the relationship between them is necessary for the development of older adult care on a global scale for three reasons. First, meeting the growing demand for older adult care is urgent. With a more serious aging trend in the global population, the demand for OACS is increasing rapidly (2). The NQPF provides strong technical and industrial support for digital OACS, while traditional OACS can overcome the limits of time and space and enhance the service accessibility through the NQPF. This allows more old people in remote areas or those with limited mobility to gain OACS with high efficiency and convenience, such as telemedicine and smart health monitoring. Second, sustained innovations and development of the older adult care industry are required on a global scale. The integration of the NQPF and OACS will generate new forms of the older adult care industry, such as older adult care big data services and smart older adult care communities. Enterprises can also research and develop innovative and competitive older adult care products and services through the effects of NQPF to promote the highly qualified development of the older adult care industry. Third, understanding the relationship between the NQPF and SOACSS is conducive to global cooperation and exchange in the older adult care industry on a global scale. Through the NQPF, the advanced experiences and technologies of digital OACS can be shared among different countries. Furthermore, using successful modes of digital OACS for reference in other countries can promote the development of smart-empowered OACS on a global scale. Hence, a comprehensive and in-depth understanding of the logical relationship between the NQPF and SOACSS is essential.

Reasonable application of NQPF in existing systems of OACS is crucial for establishing SOACSS through scientific and technological innovation. For instance, according to the Wuhou YearBook 2024 (3), the number of people over 60 was 164,800 in the Wuhou District of Chengdu in China until 2023, increasing by 9% and accounting for 23.88% of the total population. Owing to the population aging crisis, the Wuhou government launched a project called “Yijutong” to establish the smart-comprehensive information platform for community home-care services. It aims to provide older adults with a diversified and personalized OACS regardless of their location (4). Furthermore, the effects of the NQPF can promote the upgrade of the OACS through scientific and technological innovations. Older people with chronic diseases in the Wuhou District can also use smart wearable devices and home medical monitoring equipment, such as smart Bluetooth thermometers and glucometers, to collect vital sign data. Vital sign data from older individuals will be uploaded automatically to the telemedicine platform, from which doctors can check to determine their health status. This not only improves the efficiency of the OACS but also enhances service accessibility. However, despite this, the present digital system of the OACS still faces different challenges, such as the uneven quality of the OACS and low digital cognition of older people (5). Thus, this research aimed to analyze the practical effects of the NQPF on the transformation of SOACSS and to contribute to enhancing the quality of life of older adults. It is expected to serve as a valuable resource and reference for policymakers, older adult care providers, and researchers.

## 2 Literature review

### 2.1 NQPF

Productivity is the integration of human labor and production resources, such as machinery and steel, in the production process; it denotes the capability to create economic value and social wealth with four core elements: human resources, capital, science and technology, and management (6). The level of productive force is also a key index of a country’s economic development and social progress.

The NQPF serves as a new economic and societal growth model for a country with scientific and technological innovations to achieve industrial upgrades via digitalization and connectivity. It has high technology, efficiency, and quality and aligns with the new development philosophy in comparison with traditional productivity development paths that depend heavily on labor resources and energy input and consumption (7–8). The revolution and upgrade of productive forces involve processes ranging from quantitative to qualitative changes. When key and disruptive technologies achieve revolutionary technological breakthroughs and qualitative changes, the core factors of productive forces also experience new and inevitable changes and revolutions, ultimately generating NQPF. As an essential innovation and development in Marx’s productivity theory, the NQPF has fundamental characteristics, including innovation-driven development, green and low-carbon initiatives, prioritizing quality, and promoting opening and sharing (9).

Traditional economic theories, including Solow’s economic growth model and Adam Smith’s division of labor, consider labor and capital as core drivers of economic growth. These theories emphasize the significance of labor productivity and capital accumulation in productive processes (10, 11). However, these studies ignore the potential impacts of scientific and technological innovation. Paul Romer’s endogenous growth theory addresses this gap by highlighting that economic growth can be significantly driven by the internal factors of innovation, knowledge, science, and technology. Moreover, investments in human capital, knowledge, and innovation significantly affect economic growth (12). The NQPF aligns with this theory and focuses on the adoption of innovative technologies to drive productivity and economic development.

Some research has explored the NQPF in terms of its theoretical and empirical dimensions. Its theoretical dimension mainly focuses on its connotations and characteristics (13), impacts on the highly qualified development of education (14), tourism (15), agricultural construction and development (16), the oil and gas industry (17), and China’s path to modernization (7, 9). Currently, there are few empirical studies on the NQPF, such as agricultural modernization in China (18), and government subsidies for grain enterprises based on empirical evidence from listed companies in China (19). To date, to the best of our knowledge, no published study has engaged in qualitative cross-over research between the NQPF and SOACSS.

### 2.2 SOACSS

As the core factors of the NQPF, scientific and technological innovations play an important role in building the digitalized industry and SOACSS. There is a relationship between the inter-dependency and inter-promotion of NQPF and SOACSS.

#### 2.2.1 Smart empowerment

The empowerment theory, which originated in the field of psychology in the 1980s, refers to the process of changing the contexts, attitudes, and behaviors of different individuals and exploring their talents and potential (20). In the management field, the theory of empowerment stresses the delegation of top-down authority within the organization and implementation of precise resource allocation to achieve the mission and strategic objectives (21). Smart empowerment, the extension and expansion of the theory of empowerment, mainly adopts emerging (e.g., digital) technologies to create new research and development (R&D) methods, development pathways, and practical applications to enhance productive forces for individuals, enterprises, communities, and other subjects through empowerment effects (22). As NQPF carriers, a series of scientific and technological innovations, such as digitalization, have created new development pathways and tools for smart empowerment. As New Quality Productive Forces carriers, a series of scientific and technological innovations, such as digitalization, have created new development pathways and tools for smart empowerment. Under smart empowerment, the older adult care service system is also updating iteratively from traditional older adult care service system to smart-empowered older adult care service system (23, 24). The smart-empowered older adult care service system is a new and creative older adult care tool to meet diversified and personalized older adult care demands of older people through modern information technology, digital equipment, and Internet technology. It aims at enhancing the efficiency and quality of older adult care services for them. Based on the above analysis, smart empowerment is key to achieving the application of technology with digitization. Based on the above analysis, smart empowerment is key to achieving the application of technology with digitization.

The core of the OACS is to supply older adult care products and services that meet the requirements of older individuals (25). OACS plays a pivotal role in enhancing the quality of life of older people, thereby impacting their mental and physical health. The low quality of life of older adults significantly reduces their mental and physical health, ultimately leading to serious issues, such as depression and loneliness (26). Embedding digitalization in the SOACSS system can provide more efficient, more personalized, and safer services for older people through advanced information and digital technologies. Smart equipment, such as smart mattresses and smart bracelets, can help older adult care providers to remotely and precisely monitor the diverse physical and mental health indices of older people, including sleep quality, heart rate, activity level, and blood pressure. These smart forms of OACS can significantly improve the living satisfaction of older individuals (27). Furthermore, older people can access medical consultation and cure at home through telemedicine services, such as video calling and remote diagnostic technology. Telemedicine services reduce the frequency of offline hospital visits and enhance medical accessibility (28). Older people can also communicate face-to-face with other family members and friends through video calls, reducing the risk of depression and loneliness and improving their psychological and social health (29). In addition, the utilization of remote supervisory equipment, such as smart sensors and cameras, can monitor the living status of older people in real time to ensure their safety at home; when an emergency occurs, the smart equipment can immediately call preset contacts or emergency centers to provide timely medical assistance for older people (30). However, SOACSS has negative impacts. Not all older adults are proficient in using smart equipment or digital technology. Owing to the differences in age, education, and economic conditions, some cannot fully utilize these smart technologies, leading to digital divide (5), and feelings of confusion and failure, thereby reducing their quality of life (31). While digital equipment provides social opportunities and platforms for others, for older people, the long-term use of video calling technology as a kind of smart-empowered older adult care service technology might even exacerbate their sense of social isolation (32). In general, scholars’ reviews on the impact of SOACSS differ. However, there is conflicting evidence on the impact of digital older adult care.

To summarize, on the one hand, existing research on NQPF has mostly focused on its connotation, characteristics, and impacts on some fields, such as tourism and education. On the other hand, studies on smart OACS have primarily discussed its meanings and positive and negative impacts on improving older people’s quality of life. However, no attention has been paid to the methods and logic of the transformation of SOACSS through NQPF. This research explores the effects of NQPF on SOACSS and its transformation logic based on the theory of supply and demand, filling a research gap in literature. This study will serve as a reference for promoting digital transformation strategies for the OACS to enhance the quality of life of older adults on a global scale.

## 3 Methods

### 3.1 Search strategy

A stringent literature search was performed to explore how the NQPF affects the transformation of SOACSS. The comprehensive search was conducted on the Web of Science, National Knowledge Infrastructure (CNKI), Google Scholar, ProQuest, Website of the People’s Government of Sichuan Province, and the Health Technology Assessment Database (HTAD) on 7 January 2025. It focused on research and articles published in English and Chinese with reported connotations and characteristics of NQPF and impacts of digital OACS. The keywords and/or terms were “old people*,” “digitalization*,” “telemedicine,” “elderly care*,” “smart elderly care service*,” “Statistical Yearbook,” “New Quality Productive Forces,” and “empowerment*.” Boolean operators of “AND” and “OR” were used as search strings. Furthermore, we adopted a direct Google search and other appropriate reference-tracing methods to avoid missing related studies.

### 3.2 Extensive review

An extensive review and analysis of the available literature on digital older adult care and the NQPF was conducted using Web of Science, Google Scholar, ProQuest, HTAD, and CNKI. An exploration was implemented to determine (1) the relationship between NQPF and digital older adult care and (2) the effects of NQPF on the transformation of digital OACS. To provide a clear visual angle, this research presents two figures: The first analyzes the driving idea of NQPF to promote the transformation of SOACSS, and the other analyzes its transformation logic based on the supply-and-demand dimensions. Furthermore, owing to the potential ethical and privacy risks for digital industries, ethical and privacy issues, realistic dilemmas, and countermeasures are discussed in the Discussion and Realistic Dilemmas and Countermeasures sections.

## 4 Results

### 4.1 NQPF and SOACSS

#### 4.1.1 Driving idea

**Figure 1:**
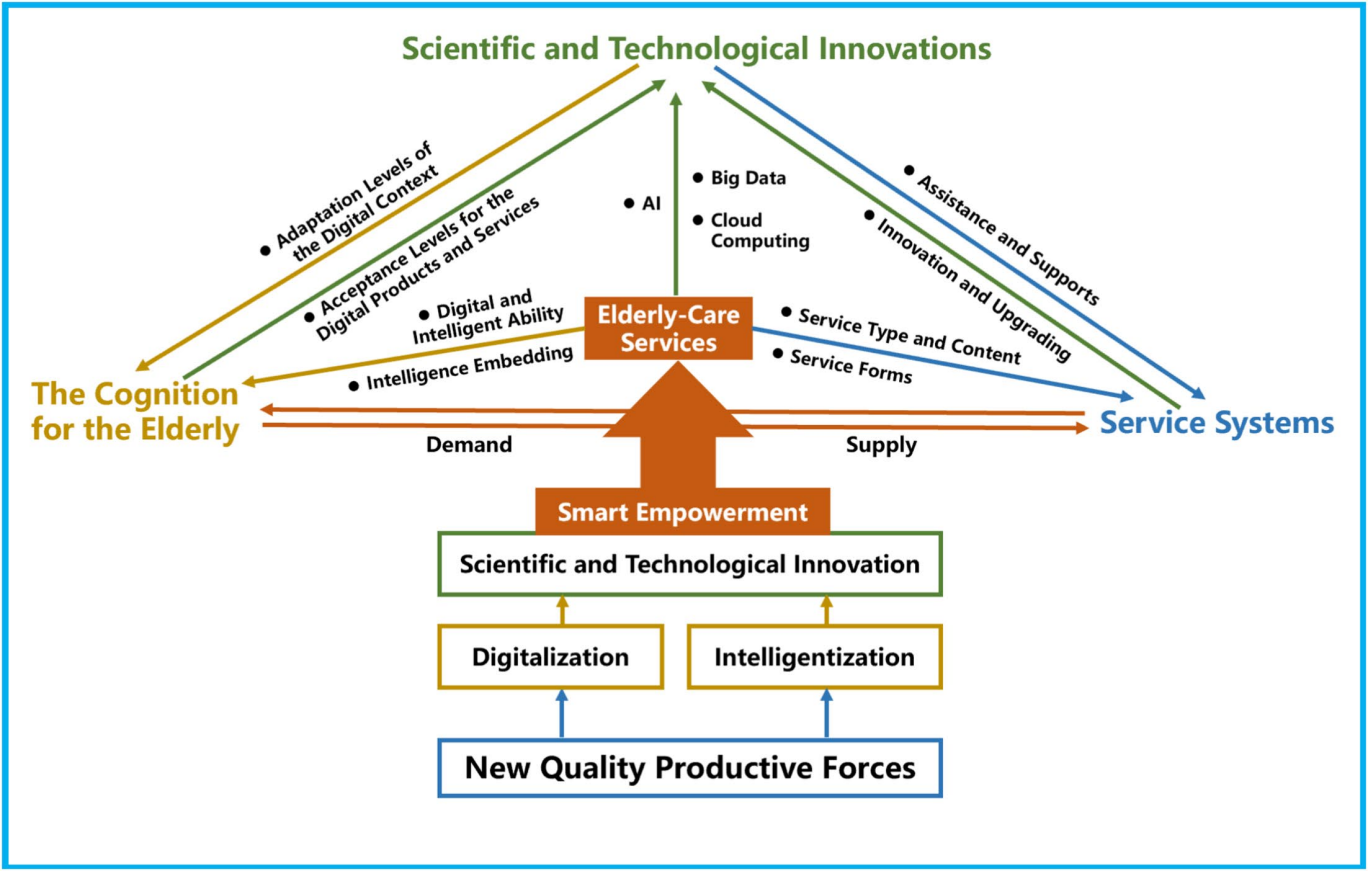
Driving idea for the transformation of smart-empowered older adult care service system via New Quality Productive Forces.

Smart empowerment is an important driving force for the NQPF to promote the transformation and upgrade of the traditional OACS. Three factors drive the interconnection and interaction between the supply and demand of SOACSS. First, scientific and technological innovation, as the carrier of the NQPF, emphasizes the integration of diversified digital information technology with the OACS to enhance its digital level, including information data collection and sharing, demand exploration, and supply of digital equipment. Second, the cognition of digitalization for older people is a key factor in determining whether older people are willing to use SOACSS, which mainly relates to demand, acceptance degree, and use intention. Third, SOACSS serves as a connecting bridge for the transformation from the traditional OACS to a digitalized service system through scientific and technological innovations such as intelligent wearable devices and telemedicine. Figure 1 illustrates the interconnections and interactions of the three factors under the NQPF to promote SOACSS upgrades.

#### 4.1.2 Promoting the transformation of SOACSS through NQPF

As a carrier of the NQPF, scientific and technological innovations can integrate digitalization into the OACS and enrich the types of OACS that can be made available to older adults, such as data collection and health monitoring. The NQPF can promote the digital transformation of OACS via smart empowerment through three factors.

The first is the establishment of an information and data management system. The NQPF emphasizes the adoption of information and digital methods to continuously improve and upgrade each information and data management step. Previously, managing the data of OACS was challenging. This is mainly due to the large volume of information and data on older people and the difficulty of sharing these among older adult care suppliers. However, with scientific and technological innovations, such as AI and cloud computing, a unified information and data management system can be established. This system provides a digital platform for older adult care suppliers to collect, store, archive, and share data and information about older people. It also reduces the complexity of information and data management. Through the digital platform, different older adult care providers can achieve tele-cooperation and accurately position older adult care demands based on the information and data. They can also allocate resources more precisely and offer personalized OACS.

The second factor enriches the types of OACS through digitalization. Through the NQPF, digital science and technologies have given rise to a new older adult care industry with abundant smart-empowered elements that promote the digital transformation of the OACS. Through scientific and technological innovation, the dependence on human labor in the traditional OACS can be significantly reduced. Currently, the OACS employs digital elements to enrich related equipment choices, including medicine delivery robots, intelligent blood pressure monitors, electronic health watches, electronic blood glucose, and lipid meters. This equipment can be used to respond to older adult care demands in a timely manner and offer diverse older adult care service types, such as supervising health indices and automatically reminding patients of health risks, which can ease the work burden on medical staff (31).

The third factor concerns measures to improve the social range, frequency, and methods used by older adults. Due to the limitations of geographical factors, equipment, and individual health, the social range and methods used for older people are limited. This means that most older adults only can frequently communicated face-to-face with others, which is not only inefficient and inconvenient but can also impact their willingness to communicate. However, under the NQPF, older adults can use digital and intelligent equipment to keep in touch with their family, friends, and doctors anytime and anywhere. The increasing number of older adult individuals using digital and intelligent equipment to communicate with others has promoted the digital transformation of the OACS through smart empowerment.

#### 4.1.3 Digital cognition and SOACSS

As digitalization is an important part of scientific and technological innovation, the degree of cognition of digitalization greatly affects the development of the OACS with smart empowerment. The capacity to use and awareness of digitalization are key factors in assessing the cognitive level of the SOACSS among older individuals. If the degree of difficulty of digital older adult care equipment surpasses that of older people, their willingness to use this equipment and OACS with smart empowerment will inevitably largely decrease due to the inherent laziness of human beings. This also has a profound effect on older adults’ acceptance of smart empowerment in OACS.

Based on the theory of social cognition, there is a strong correlation between the intelligibility of new ideas, scientific and technological innovation, and the attitudes and perceptions of related information for human beings. In this case, there is a direct correlation between the cognition of the OACS, the smart empowerment of older people, and the perceptions and understanding of related data and information (5). When SOACSS can meet the basic values and demands of older adult care service selection for older people, the acceptance and popularization rates of OACS with smart empowerment will increase continuously, owing to its high convenience, efficiency, science and technology, and quality. However, some older people cannot keep up with the pace of scientific and technological development (33), causing their digital cognition to decrease. With the popularity of digital services, such as online registration and remote outpatient services, some may already possess considerable digital awareness and ability, extending from the pure pursuit of basic OACS to smart older adult care projects such as online social networking and telemedicine. However, if those lacking sufficient digital cognition still do not have any awareness or motivation to improve their digital ability and awareness, it will inevitably be a barrier to the transformation of OACS with smart empowerment.

#### 4.1.4 Service system and digitalization

OACS is a general term used for situations in which suppliers deliver various types and forms of OACS. Through scientific and technological innovations, the carrier of the NQPF, digitalization can be integrated into the service system, finally forming the SOACSS. The SOACSS promotes the improvement and upgrade of the OACS in the following three aspects.

First, SOACSS offers a digital platform for different older adult care suppliers and forms a multi-subject coordinated joint participation mode. They can be used to transform traditional OACS delivery channels from offline face-to-face OACS to online OACS, including telemedicine, online healthcare consulting, and remote health-keeping guidance services, enhancing the accessibility, convenience, and efficiency of the OACS.

Second, the SOACSS provides a clear and digital operational platform for older adult care suppliers to conduct coordinated integration and rational allocation of older adult care resources. Through scientific and technological innovations, older adult care providers can adopt advanced intelligent older adult care service systems to integrate and classify the different older adult care demands online. They can also coordinate and allocate resources of OACS rationally and precisely based on older people’s actual older adult care demands, effectively reducing resource waste. The NQPF conforms to the concept of resource-coordinated allocation, concentrating on the highly efficient utilization of older adult care resources, which is an important method for constructing the SOACSS with high quality, high technology, and low resource consumption.

Third, through the SOACSS, family, friends, and older adult care institutions can overcome the limitations of space barriers by offering OACS and healthcare services to older people anytime and anywhere, such as remote healthcare reviews and supervision of health indices. Simultaneously, when older adult people accept OACS in older adult care institutions, they can utilize diverse digital equipment, such as intelligent monitors and sensors, to access uninterrupted remote health, safety monitoring, and environmental control. Older adult care institutions can warn older adults about health risks in advance and arrange manual intervention measures, enabling OACS institutions to continuously provide older adults with medical control, diet management, and rehabilitation support to ensure safety and quality.

### 4.2 Transformation logic

Scientific and technological innovations, as carriers of the NQPF, are integrated into the OACS to generate the SOACSS. The OACS with smart empowerment can adopt advanced digital equipment to address a series of issues faced by the present system of OACS in the supply dimension, including staff shortages, service quality differences in different regions, limited-service types, and low service accessibility. The transformation of OACS with smart empowerment not only enhances its efficiency and quality but also meets the diversified and personalized older adult care demands. The NQPF can promote the digital transformation of older adult care service systems with smart empowerment via both the supply and demand dimensions.

Providing a higher-quality OACS is the basic aim of transforming the SOACSS. Previously, there was no unified system of OACS to clarify the division of responsibilities for different older adult care suppliers, hindering them from co-operating effectively. However, under the NQPF, a smart-empowered older adult care service platform can be established and used to distribute work responsibilities to older adult care suppliers. Older adult care providers can co-operate to form a multi-subject coordinated mode to promote the SOACSS transformation.

#### 4.2.1 Supply dimension

Four entities support the transformation of the SOACSS into the supply dimension. The first is the government. Through overall top-to-bottom planning, the government enacts policies to promote the transformation of SOACSS and provide financial support. In addition, through the NQPF, the government adopts advanced science and technologies to build the platform of OACS, enriching the channels and methods of older adult care supply.

The second category comprises enterprises. Enterprises research and manufacture older adult care products and equipment based on actual older adult care demands through the NQPF’s factors with the high technology and the R&D. With the increasingly abundant choice of digital older adult care products and equipment, enterprises can now meet the demands of high-qualified services, more service types, and smarter older adult care environments.

The third is community service centers. Community service centers adopt SOACSS to collect, store, and archive data and information on older people and directly share these on the platform of OACS with other older adult care suppliers. Then, by cooperating with the suppliers, they precisely analyze and position older people’s care demands based on their information and data on the platform of OACS to allocate older adult care resources.

The fourth is family. Young people in the family teach older people to improve their digital cognition through digitalization education. Enhancing the frequency of communication regarding the use of digital products in daily life can motivate the growing older population to integrate into the digital environment and use smart-empowered OACS efficiently, scientifically, safely, and reasonably.

#### 4.2.2 Demand dimension

The final objective of the transformation of SOACSS through the NQPF is to comprehensively meet the care demands of older people. However, older adults have varying demands for smart-empowered OACS owing to different digital cognitions, self-characters, social identities, income levels, and health statuses. These differences affect the selection of older adult care products or services, judgment methods, and cognitive standards (34).

There are two main methods to promote the transformation of SOACSS in the demand dimension.

First, the collection and analysis of information and data on older adults using innovative information and data management systems can precisely determine the actual demands of OACS. At present, with the trend of high-speed iterative updating on the supply side in the OACS market, SOACSS faces the issue of supply exceeding demand. This is because most older people cannot sufficiently position their older adult care demands and select appropriate digital products and equipment. Under the effects of the NQPF, advanced science and technologies, such as innovative information and data management systems, can be used to precisely analyze and position the demands of the OACS intelligently and allocate older adult care resources rationally.

Second, the cognition of smart empowerment and the acceptance of smart-empowered OACS by older people serve as the connecting hubs to achieve the co-ordination of its supply and demand. Owing to differences in intellectual literacy, older adults have different levels of cognition and acceptance of digitalization. Many older adults cannot effectively integrate into the digital environment because of a lack of awareness regarding smart empowerment (35). For instance, using an electronic map to find a destination remains challenging for old people in China. However, those who can integrate into the digitalized environment can use digitalized older adult care products and equipment, such as telemedicine systems and smartphones. Thus, improving the public’s cognition and digitalization ability is essential for transforming the SOACSS.

#### 4.2.3 Cross-collaboration between supply and demand

The key to promoting the transformation of SOACSS is the cross-collaboration between supply and demand to form a dynamic balance. In the supply dimension, transforming SOACSS requires a clear division of responsibilities so that older adult care suppliers can collectively form a multi-subject coordination mode. In the demand dimension, to enhance the demand for smart-empowered OACS, the cognition and digitalization ability of older people need to be improved so that they can effectively integrate into the digitalization environment, which can gradually enhance the popularity of smart-empowered OACS.

**Figure 2:**
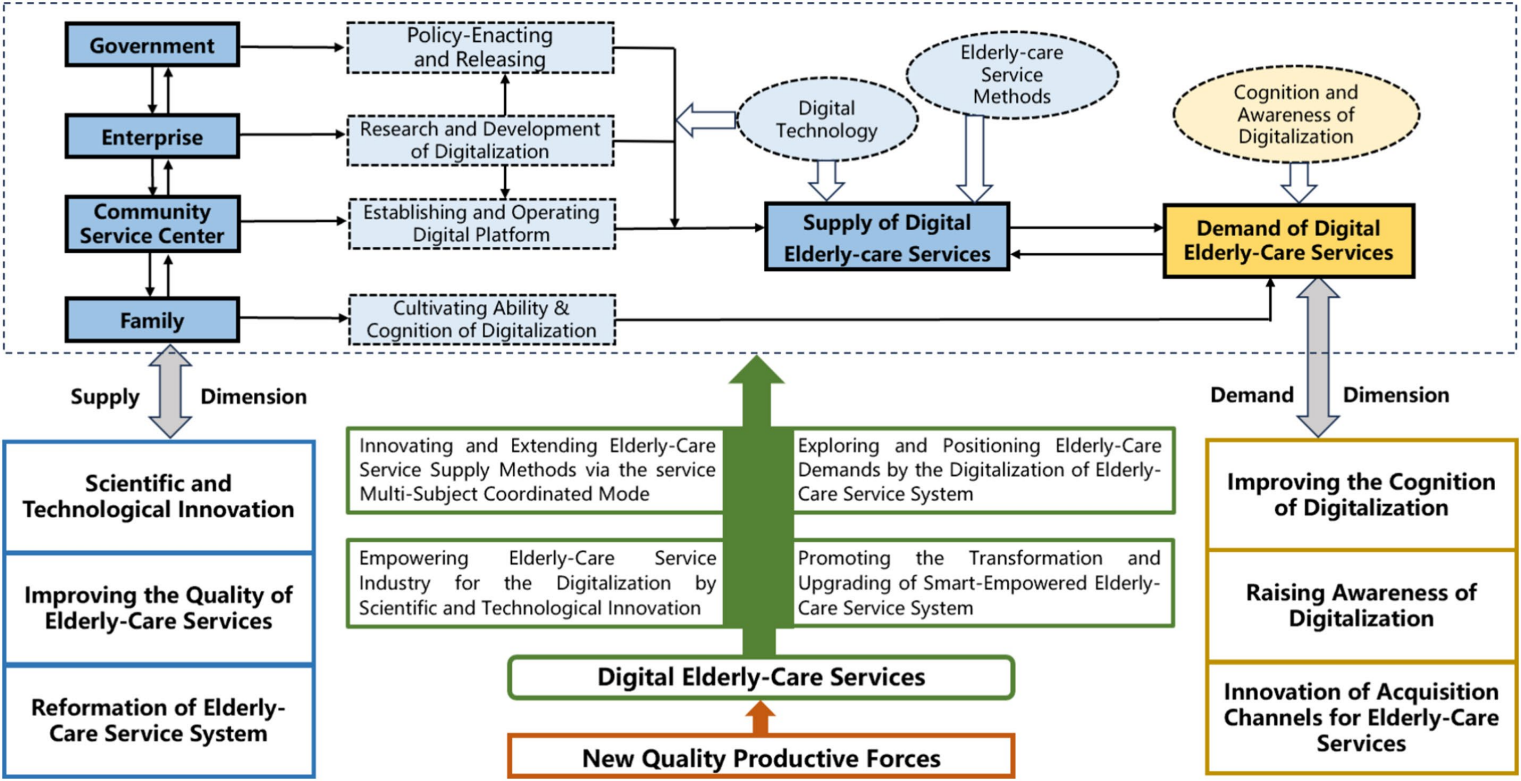
Transformation logic of smart-empowered older adult care service systems through New Quality Productive Forces.

The demand dimension has traction effects on the supply dimension, which can improve the precision of the supply for the OACS. This is a process with a mutual impact; the supply dimension needs to research, develop, and produce smart-empowered older adult care products and equipment according to the actual demands of older people. Meanwhile, the demand dimension needs to adapt to the functions and attributes of smart-empowered older adult care products and equipment researched and developed in the supply dimension. Figure 2 illustrates the transformation logic of a SOACSS in terms of supply and demand.

## 5 Discussion

### 5.1 Ethical and privacy challenges of the digitalization for OACS

Digitalization of the OACS has become an important method for improving the quality of life of older individuals (36). Digital science and technologies, such as telemedicine and big data analysis, not only improve the efficiency of OACS but also provide more personalized and convenient services for older people. However, its application inevitably leads to ethical issues and privacy challenges, including data privacy and safety, social isolation, technological exclusion, and the ethical responsibilities of using digital equipment. This section explores the ethical and privacy challenges of the digitalization of the OACS and how they integrate into future policy recommendations.

First, smart equipment and systems store vast amounts of basic health data and information about older people. If these are mishandled or leaked, the privacy safety of them will be seriously threatened. In the future, governments should enact and implement more stringent data and information protection regulations to enhance their supervision and protection. Advanced encryption technology should be used to protect data and information from unauthorized access.

Second, although digital platforms can provide older people with opportunities for online social networking, virtual social networking cannot completely replace face-to-face communication. Using it for a long time will cause and even exacerbate a sense of social isolation among older people. In the future, society should organize diversified social opportunities and develop online-and-offline social networking mode for older people.

Third, when the digital system of the OACS malfunctions due to various issues, such as health monitoring equipment failure or algorithmic decision-making errors, the related attribution of liability may be unclear. In the future, a clear ethical standard should be established to guarantee fairness and transparency in the decision-making process. In addition, each older adult care supplier’s responsibilities should be clearly distinguished. When ethical issues arise, the government can hold related parties accountable for these issues.

Fourth, owing to the different demands of the OACS, the R&D and application of the SOACSS may overlook some older people’s demands, leading to unfairness. In the future, the R&D of SOACSS should consider the different demands and backgrounds of older people, including culture, languages, health, and financial conditions.

The ethical issues of digital OACS involve various aspects, such as privacy protection, fairness, and responsibility attribution. Addressing these issues requires multidisciplinary efforts, including those of technology developers, ethicists, policy makers, and social workers. The future development of smart OACS should find a balance between technological innovation and ethical constraints to provide older adults with a higher quality of life.

## 6 Conclusion

### 6.1 Realistic dilemmas and countermeasures

#### 6.1.1 Dilemmas

SOACSS aims to address the social issues of the aging population and meet the growing needs of older adults for a better life. Through the NQPF, the traditional system of OACS can integrate scientific and technological innovations to digitalize its innovative service methods and improve service quality. However, three obstacles hinder the transformation of SOACSS.

The first one is the uneven level of SOACSS development, which is due to significant differences in the economic development levels of different regions. The level of economic development is a significant factor in the transformation of SOACSS as the scientific and technological innovations used for the R&D and construction of SOACSS incur significant costs in the early stage. This similarly applies to the procurement, production, and maintenance of OACS products and equipment in the later stage. However, there are significant regional differences in China’s economic development. Eastern China is characterized by high-quality economic development, with a large amount of capital invested in R&D for the transformation of SOACSS. In comparison, for the economically backward regions in central and western China, which lack sufficient support for finance, labor, and digitalization, the efficiency and construction progress of SOACSS are low and slow. This results in the polarization of digital cognition for the public between eastern China and central and western China. This can also be attributed to the difference in the popularity of digitalization, which is a barrier to the transformation of SOACSS.

The second concerns the weak application capacity of scientific and technological innovations. The application level of scientific and technological innovations can reflect a country’s ability to transform R&D achievements in science and technology into practical applications. Appropriate application of scientific and technological innovations is a basic condition for promoting economic development, improving the public’s quality of life, and strengthening a nation’s core competitiveness. Recently, China has gradually made breakthroughs in the R&D of digital science and technology, and various high-end science and technologies have emerged. For instance, during the coronavirus pandemic, distance education and telemedicine have developed rapidly in China. Through Internet technology, distance education and telemedicine can cover a wider population, especially those in remote areas (37, 38). However, most older adults in China have poor digital cognition and are unfamiliar with smart-empowered older adult care products and equipment. This leads to a series of technical difficulties in the application of OACS, such as ensuring security in the use of digital older adult care devices and the irregular use of methods for smart-empowered older adult care products and equipment. A weak application capacity for the utilization of scientific and technological innovation impedes the transformation of SOACSS.

The third dilemma is the imperfect staff-training system for the smart-empowered OACS. A perfect staff training system is the basis of highly qualified service ability for smart-empowered older adult care staff and can guarantee the effective operation of SOACSS. High-quality staff for smart-empowered OACS is essential for the transformation of SOACSS. However, the number of staff in the digitalized OACS remains limited, which can be attributed to the following two aspects: The first is a lack of sufficient training time. At present, most older adult care service staff cannot master new digitalization abilities for the OACS in the short term, such as the back-office operation of SOACSS and the management of digital products and equipment of the OACS. Moreover, after the staff receive digitalization training on the supply of the OACS, the full impact of the training effect will not be immediately visible, owing to the lag in the training effect. The second aspect concerns the training costs. Staff training for the supply of smart-empowered OACS must cover the practical operation and control of digitalized OACS. Thus, comprehensive procurement of older adult care products and equipment for digitalization is required in all regions of China to support staff training in smart-empowered OACS. Simultaneously, to provide highly qualified staff training for the supply of smart-empowered OACS, experts in digitalized OACS must be acquired, which increases the budget. Currently, China’s level of economic development is insufficient to support the construction of SOACSS in all regions.

#### 6.1.2 Countermeasures

There are five main countermeasures to address these dilemmas in the process of transforming SOACSS.

First, a comprehensive understanding and precise positioning of older adult care needs for older people is the basis for supplying the smart-empowered, high-quality OACS. Supply and demand must be considered to reduce the ineffective consumption of older adult care resources. Through the NQPF, a series of advanced technologies, such as AI and big data, can be used to collect and archive data and information on older people of different ages, income levels, and health conditions. Advanced technologies, such as cloud computing and intelligent robots, can be used to analyze and precisely position older adult care demands automatically for older people and subsequently provide the OACS on demand.

Second, the publicity of smart empowered OACS should be increased through the penetration of scientific and technological innovations. We should adopt diverse science and technologies, such as the Internet and AI, to enable the public, especially older individuals, to comprehensively understand smart-empowered OACS and improve their popularity among the public. Currently, the number of older people who use digitalized OACS is low. However, after knowing the functions, principles, and methods of application for smart-empowered older adult care products and equipment and the range of applications of smart-empowered OACS, a gradually increasing number of older people will be willing to try smart-empowered OACS.

Third, the level of digital cognition in older adults is a key factor in promoting the transformation of SOACSS. Older adults should be trained in the application of digitalization, enabling them to use older adult care products and equipment conveniently and safely. Simultaneously, older adults should be encouraged to use digital products and equipment for communication, entertainment, and studying. With a sustainable increase in proficiency in the utilization of digital products and equipment, the digital cognition of smart-empowered OACS for older people could be greatly improved, helping them integrate into the digital environment.

Fourth, establishing a sound staff-training system is crucial for supplying various highly qualified, smart-empowered OACS. Policies should be enacted for talent-training planning for smart-empowered OACS. Talent-training planning is divided into four steps: initial, growth, medium, and maturation. The goal of the initial stage is to comprehensively understand SOACSS. The growth stage aims to facilitate the easy use of older adult care products and digitalized equipment. The medium stage seeks to use diverse older adult care products and equipment for expert-level digitalization. Finally, the maturation stage aims to supply the highly qualified smart-empowered OACS to older people through diverse products and digital equipment. Talent-training planning should accurately position the training stage, so the trainee can study according to the corresponding stage. In addition, through scientific and technological innovation, we should utilize AI, big data, and cloud computing to record and store training and teaching content in the form of videos concerning smart-empowered OACS. Trainees can watch these videos to study and review the content anytime and anywhere, which can improve their ability to supply the smart-empowered OACS.

Fifth, implementing an economic compensation policy is essential for promoting the balanced development of the smart-empowered OACS for different regions. Measures should be taken to form a coordinated development pattern both upstream and downstream of the smart-empowered older adult care service industry. This includes strengthening market supervision and the top-level design of SOACSS, promoting the sinking of high-quality digital resources, and sharing digital technology and resources with different regions. Simultaneously, the government also plays a core role in this process. It can invest more in financial support to strengthen economic subsidies to counties, grass-roots areas, and other economically underdeveloped regions, promoting the construction and transformation of SOACSS in these regions. In addition, governments should establish core older adult care service stations in economically underdeveloped regions of China. These stations can cover their respective sub-regions and grass-roots areas and aid in the distribution of resources while ensuring quality management and regular evaluation of smart-empowered OACS to promote the SOACSS transformation.

### 6.2 Contributions

Grounded in the theoretical framework of supply and demand, this study presents an idea for understanding the integration of smart empowerment and the OACS. Based on the effects of the NQPF, this research provides important insights into the transformation of SOACSS around the interaction effects of scientific and technological innovations, digital cognition, and service systems. It also reveals how the NQPF promotes the transformation of SOACSS, as shown in Figure 1. Overall, the digitalization embedded in the OACS enriches older adult care types and content and improves the quality of life of older adults. The results shed light on the potential patterns of integrating smart empowerment into the OACS.

In addition, this research illustrates the transformation logic of SOACSS through the NQPF, based on supply-and-demand dimensions. The results underscore the interaction between the supply and demand for older adult care, highlighting the importance of the multi-subject coordinated mode in the SOACSS. The results can contribute significantly to the upgrades of older adult care policies and enhancing the work coordination efficiency of older adult care suppliers.

### 6.3 Limitations and future research directions

Despite the exploration of the transformation logic of SOACSS, this study has certain limitations. First, it depends solely on peer-reviewed published attention and literature, descriptions of transformation logic, and theoretical analysis, ignoring empirical data support. Second, the review did not combine specific situations in all regions and cultural contexts for a concrete discussion and analysis. There may be significant differences in the acceptance, culture, living habits, and social support of older adults in different regions. These differences may affect the characters and solutions to ethical and privacy issues. Addressing these limitations in the future will offer more comprehensive and nuanced insights into the development of SOACSS.

Hence, future studies should aim to overcome these limitations and dilemmas regarding the digital development of the OACS, such as ethical and privacy challenges, to promote the digital transformation of OACS. It can enhance the quality of life of older adults. This may require the adoption of more empirical studies with large-scale data collection and analysis in cross-cultural regions. Through this method, older adult care suppliers can determine the actual demands and experiences of older individuals when using digital OACS. They can also compare the ethical and privacy challenges of older people from different educational, financial, sexual, and age backgrounds when using the smart-empowered OACS. This is helpful for the government when testing existing theoretical hypotheses and providing data support to enact digitalization policies. Simultaneously, an evaluation mechanism for new science and technology should be established to conduct regular assessments of its effects on ethics and privacy. The evaluation mechanism can integrate the latest science and technology into ethical considerations in a timely manner. Furthermore, various digital technologies integrating into the older adult care would gradually replace older adult care staff. Older people would have to face with and even accept new digital older adult care models. This causes new challenges of older people for their digital cognitions, digital abilities, adaption abilities, and learning abilities. In the future, the vast potential of smart-empowered technologies to improve the quality of the OACS and enhance the quality of life of the older people warrants further exploration.
